# Evaluation of Quality Control Methods for Foot-And-Mouth Disease Vaccines by High-Performance Liquid Chromatography

**DOI:** 10.3390/pathogens9030194

**Published:** 2020-03-05

**Authors:** Mun-Hyeon Kim, Seon-Jong Yun, Yeon-Hee Kim, Hyang-Sim Lee, Ji-Yeon Kim, Ji-Ye Kim, JeongWoo Kang, Yong-Sang Kim, Min-Goo Seo

**Affiliations:** Animal and Plant Quarantine Agency, Gimcheon, Gyeongbuk 39660, Korea; kmh8110@korea.kr (M.-H.K.); paru33@korea.kr (S.-J.Y.); vetyh@korea.kr (Y.-H.K.); leehs76@korea.kr (H.-S.L.); jiyekim@korea.kr (J.-Y.K.); yskim0621@korea.kr (Y.-S.K.)

**Keywords:** 146S antigen, evaluation, foot-and-mouth disease, high-performance liquid chromatography, national lot-release testing, vaccine, validation

## Abstract

Foot-and-mouth disease (FMD) is considered one of the highly contagious viral infections affecting livestock. In Korea, an FMD vaccination policy has been implemented nationwide since 2010 for the prevention and control of FMD. Since the vaccines are imported from various countries, standardized quality control measures are critical. In this study, we aimed to validate a high-performance liquid chromatography (HPLC) device in the Animal and Plant Quarantine Agency lab and identify an appropriate FMD vaccine pretreatment method for HPLC—a simple, reliable, and practical method to measure antigen content. Based on the analyses of specificity, linearity, accuracy, repeatability, intermediate precision, limits of detection, and limits of quantification using FMD standard samples, we validated the method using a standard material. Overall, we confirmed that the HPLC technique is effective for the quantitative assessment of the FMD virus 146S antigen in Korea. Using commercial FMD vaccines, we evaluated three separation methods and identified the method using n-pentanol and trichloroethylene as optimal for HPLC analysis. Our HPLC method was effective for the analytical detection of the antigen content in FMD vaccine, and it may be useful as a reference method for national lot-release testing.

## 1. Introduction

Foot-and-mouth disease (FMD) is a contagious viral disease affecting cloven-hoofed animals, including pigs, goats, cattle, buffalo, and sheep. It is initially characterized by high fever that rapidly declines after 2–3 days, followed by vesicles in the feet and oral mucosa [1]. Although mortality is normally low in adult animals, FMD outbreaks result in significant economic losses due to production loss and the subsequent restrictions on the global trade of live animals and their products [2].

The FMD virus is in the genus *Aphthovirus* belonging to the family Picornaviridae. Its genome is a single stranded, positive-sense RNA molecule of 8.5 kb with a single open reading frame. There are seven known serotypes with distinct immunological properties, namely, O, Asia 1, C, A, South African Territories (SAT) 1, SAT 2, and SAT 3 [3]. The intact virion contains four specific particles—the antigenic 146S, empty capsid 75S, virus infection-related peptide 45S, and protein subunit 12S. Most FMD vaccines consist of inactivated virions, and their quality is primarily determined by the 146S virus particle content. The efficacy of inactivated FMD vaccines is also dependent on the intact capsid and 146S particle sedimentation rate [4,5].

Eleven FMD outbreaks have been reported in South Korea between 2000 and 2019 [6]. Vaccination is the most practical and effective means of controlling or preventing these outbreaks, and a national vaccination policy was established for all FMD-susceptible animals in 2010. Vaccines are imported from the United Kingdom, Argentina, and Russia. In this milieu, antigen quality control is critical. Besides 146S quantification, evaluation of efficacy, safety, sterility, identity, purity, potency, stability, and immunity contributes to the quality control process. In South Korea, the Animal and Plant Quarantine Agency (APQA) supervises the continuous quality control process of imported FMD vaccines.

The development of a high-performance liquid chromatography (HPLC) assay as an alternative to animal testing has been the focus of several studies [7,8,9,10,11,12]. HPLC is a rapid, simple, and reliable separation method, wherein size-exclusion chromatography (SEC) is used to analyze vaccine constituents. HPLC has been used to test influenza vaccines [8] and the 146S content in FMD vaccines [9]. A vaccine manufacturer in Argentina—Biogénesis Bagó S.A.—has developed a gel permeation chromatography method for FMD virus antigen quantitation based on SEC [10]. SEC is commonly used to monitor the purity of products in biopharmaceutical industries and is independent of buffer pH, ionic strength, and particle charge, making it an appropriate analytical technology across a range of process streams. In 2011, a proprietary HPLC method based on the SEC separation principle was developed, validated, and patented by Biogénesis Bagó S.A. [11]. It analyzed the specificity, repeatability, intermediate precision, accuracy, linearity, limit of quantitation (LOQ), limit of detection (LOD), robustness, and analytical sample stability; it also performed concordance analysis with ultracentrifugation 146S results [12].

An automated HPLC method for the measurement of FMD virus antigen content has the potential to eliminate the need for animal testing [12]. Therefore, the goal of our study was to develop a quality control method for the FMD vaccine without using animals, with the aim of validating the HPLC device in the APQA lab and identifying an appropriate vaccine pretreatment method for HPLC. To apply the HPLC method in APQA lab, we first validated the HPLC device in our lab. In Korea, FMD vaccines are imported from three different companies; hence, there is a need for pretreatment of each sample for HPLC. Currently, there is a lack of comprehensive studies on separation methods for determining the antigen content in different FMD vaccines by HPLC. To identify an appropriate pretreatment method of each vaccine for HPLC, we investigated three methods to analyze FMD vaccines, with varying ingredients, from different manufacturers. We selected a promising, HPLC-enabled, quantitative method for the 146S antigen for national lot-release testing. It is important to evaluate the correlation between the detected quantities of FMD 146S antigen by HPLC and potency data against the virus neutralization test (VNT) values after animal vaccination. If we succeed in establishing this approach, vaccine quality can be accurately assessed by simply measuring antigen content in the FMD vaccine by HPLC alone. Therefore, finally, we aimed to develop an optimized quality control method for the evaluation of FMD vaccine potency by HPLC.

## 2. Results

### 2.1. Application of Test Methods

**Figure 1:**
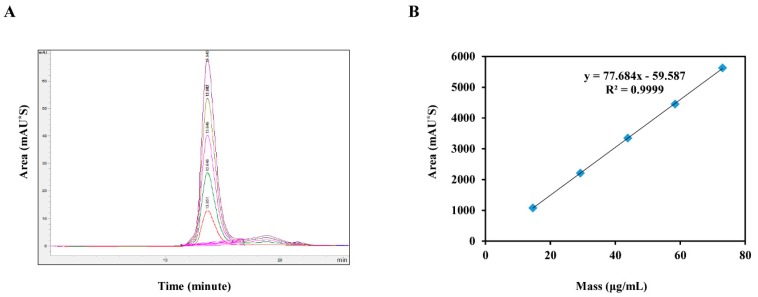
High-performance liquid chromatography (HPLC) chromatograms for the Foot-and-mouth disease (FMD) vaccine standard at 73, 58.4, 43.8, 29.2, and 14.6 µg/mL. (A) Overlay of peaks and (B) linearity of the response curve constructed using the FMD standard by HPLC.

To evaluate the specificity of HPLC for analyte detection in the presence of other components, we compared representative chromatograms of samples with potential interference of the mobile phase with those of test samples. We did not detect any interference of impurities in reagents. To establish linearity, a standard material (730 µg/mL) of five concentrations (73, 58.4, 43.8, 29.2, and 14.6 µg/mL) was prepared. We observed good linearity (*r^2^* > 0.9999) in the concentration range of 14.6–73 µg/mL (Figure 1). Repeatability was assessed by analyzing three replicate samples at five concentrations on the same day. The coefficient of variation (CV) was less than 1.11%, meeting the criterion for repeatability (<10%) (Table 1). Accuracy was evaluated by preparing samples spiked with known amounts of the standard in triplicate at five different concentrations to measure analyte recovery. The criterion for accuracy was a recovery rate of 85%–115%. The mean recovery rate at all concentrations was higher than 86% (Table 1).

To evaluate intermediate precision, we performed experiments using the same machines on different days. The standard material at five concentrations was analyzed three times on the same day (intra-day variation) and across a 7-day interval (inter-day variation). The %CV values for intra-day precision and inter-day precision were <2.0% and <4.0%, respectively (Table 2), satisfying the acceptance criterion (<10%). The LOD and LOQ were also calculated based on the standard deviation of response and the slope obtained from a linear plot of the standard. The LOD and LOQ values were 0.57 and 1.74 µg/mL, respectively (Table 3).

HPLC parameters, including column selection, mobile phase, maximum pressure, run time, flow rate, and absorbance, were optimized for vaccine analysis in South Korea (Table 4). The results revealed that the HPLC technique is applicable to samples in South Korea and can be used to quantify the FMDV 146S antigen content.

### 2.2. Pretreatment of FMD Vaccines

To measure the antigen content in commercial vaccines consisting of various adjuvants, we investigated pretreatment methods applicable to HPLC. For HPLC detection, the samples were treated with Benzonase after the addition of each reagent. FMD vaccines from three companies (C1, C2, and C3), comprising three different batches of O+A+A serotype vaccine from C1, two different batches of O serotype vaccine from C2, two different batches of A serotype vaccine from C2, four different batches of O+A serotype vaccine from C2, and seven different batches of O+O+A serotype vaccine from C3, were evaluated for antigen content and recovery rate by HPLC (Table 5 and Table 6). Each vaccine from the three companies was separated using the following three methods: chloroform (M1), butanol (M2), and n-pentanol and trichloroethylene (M3). The antigen content was subsequently measured by HPLC. Additionally, the antigen content in the vaccine from C1 was measured by sucrose density gradient (Table 5). We performed three trials for each vaccine batch. The %CV values for each vaccine batch were <3.0% (Table 5 and Table 6). The detected recovery rate of C1 vaccine determined by both HPLC and the sucrose density gradient was higher for M3 than for both M1 and M2 (Table 5).

**Figure 2:**
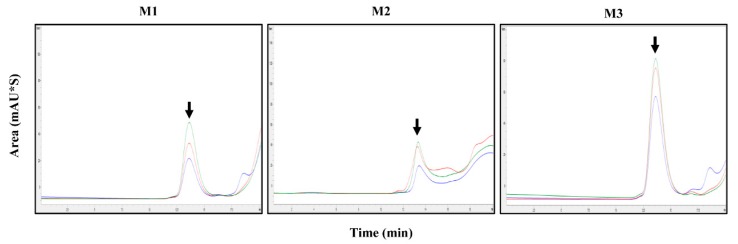
Chromatographic analysis of the C1 vaccine (different batches) against serotype O+A+A. M1, M2, or M3 was used for separation, followed by 146S antigen quantification by HPLC. Black arrows indicate measured peaks.

C1 produces vaccines against serotypes O+A+A. We analyzed these vaccines individually by HPLC after pretreatment with various methods. For serotype O+A+A vaccine, M1 yielded an average antigen content of 30.2 μg/mL, M2 yielded 22.7 μg/mL, and M3 yielded 57.9 μg/mL (Figure 2). With the sucrose density gradient method, M1 yielded an average antigen content of 34.8 μg/mL, M2 yielded 25.2 μg/mL, and M3 yielded 46.8 μg/mL (Table 5).

**Figure 3:**
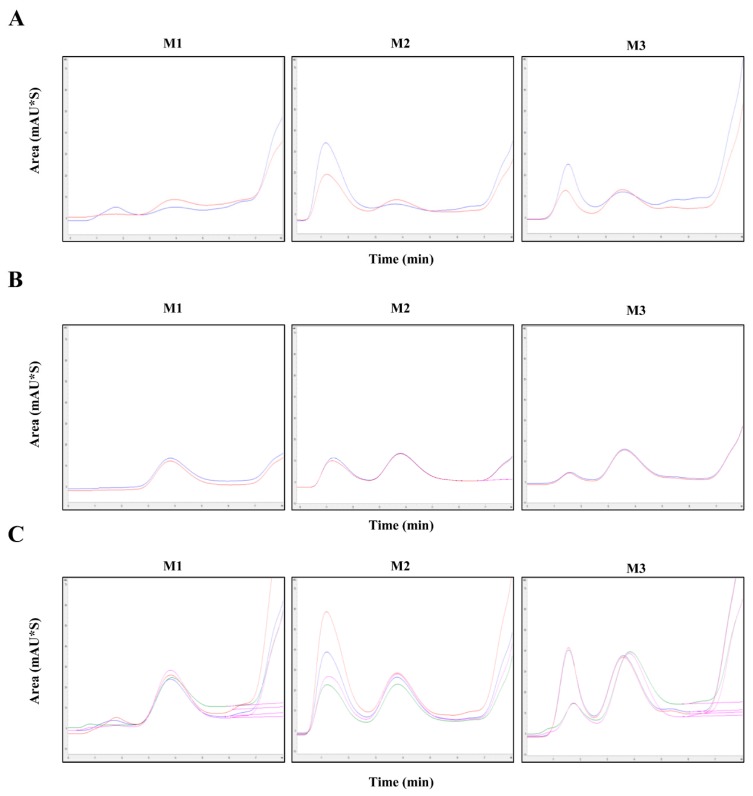
Chromatographic analysis of different FMDV vaccine strains. Chromatograms of vaccines (different batches) against (**A**) serotype O, (**B**) serotype A, and (**C**) serotype O+A from C2 were measured by HPLC after separation using M1, M2, or M3. Black arrows indicate measured peaks.

Company C2 produces vaccines against serotypes O, A, and O+A. We analyzed these vaccines individually by HPLC after M1, M2, and M3 pretreatment. For serotype O, M1 yielded an average antigen content of 3.2 μg/mL, M2 yielded 3.4 μg/mL, and M3 yielded 6.7 μg/mL. The same three methods were applied to the vaccine against serotype A. M1 yielded an average antigen content of 12.3 μg/mL, M2 yielded 12.0 μg/mL, and M3 yielded 14.0 μg/mL. For serotype O+A, the average antigen content obtained using the three methods was 20.4, 18.2, and 25.8 μg/mL, respectively (Figure 3).

**Figure 4:**
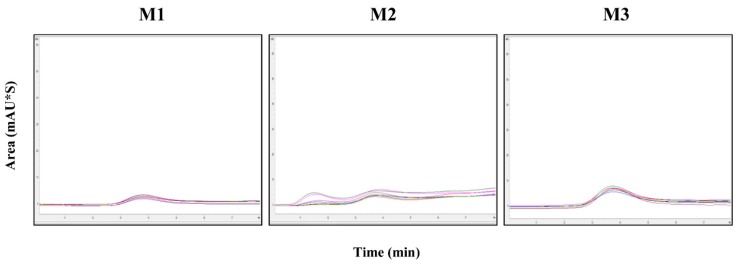
Chromatographic analysis of the C3 vaccine (different batches) against serotype O+O+A. M1, M2, or M3 was used for separation, followed by 146S antigen quantification by HPLC. Black arrows indicate measured peaks.

Company C3 provided a serotype O+O+A vaccine. The average antigen contents obtained with M1, M2, and M3 were 2.6, 2.4, and 7.0 μg/mL, respectively (Figure 4).

### 2.3. Correlation Coefficient Analysis with Sucrose Gradient Centrifugation

**Figure 5:**
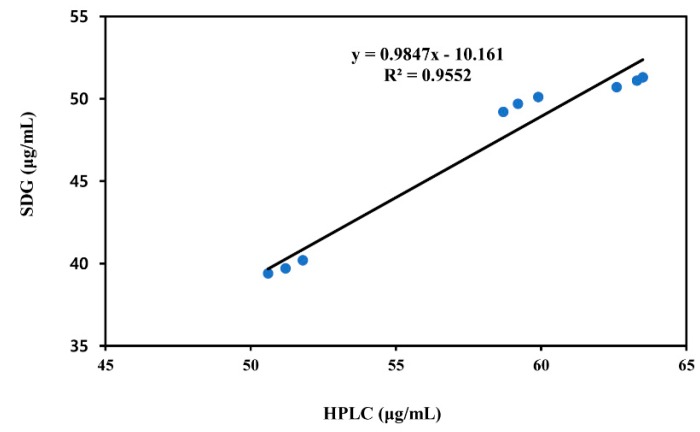
Correlation coefficient plot for HPLC and 146S results for method comparison.

Correlation coefficient analysis showed good agreement between HPLC and sucrose gradient centrifugation method in three different batch vaccines from C1 company separated by M3 (Figure 5). The correlation coefficient for all the samples considered together was 0.9773 (*r^2^* = 0.9552), with a 95% confidence interval of 0.8925‒0.9954. 

## 3. Discussion

South Korea has adopted an FMD vaccine diversification import policy and has been using vaccines imported from the United Kingdom, Argentina, and Russia. Therefore, quality control of each vaccine batch is essential to ensure efficacy and safety. In South Korea, continuous national lot-release testing is conducted in accordance with the “Korean Standard Assay of Veterinary Biological Products” by the APQA [13]. To measure the effectiveness of a vaccine, VNT is currently the gold standard for vaccine antibody titers in vaccinated animals [14]. Laboratories working with the FMD virus must have robust biosafety measures in biosafety level 3 (BL3) facilities to prevent the inadvertent release of the virus into the environment [15,16]. Therefore, it is not feasible to routinely estimate vaccine potency using VNT in non-BL3 laboratories. In South Korea, the national lot-release testing of FMD vaccines is achieved using the VNT with serum samples from vaccinated animals in the laboratory equipped with BL3. However, antigen-negative animals are not available in Korea because of the nationwide FMD vaccination strategy. Because of ever-growing use and approval of imported vaccines in the domestic market, it is critical to apply an appropriate vaccine qualification system for national lot-release testing in Korea, in order to develop a new standard for the quality verification of each vaccine. Furthermore, any new evaluation method must address animal welfare issues and financial concerns.

Therefore, the goal of our study was to develop a quality control method for the FMD vaccine without using animals, with the aim of validating the HPLC device in the APQA lab and identifying an appropriate vaccine pretreatment method for HPLC. Because vaccines from different companies have different antigen levels, there is a need for individual criteria for specific antigen level against the VNT level. In the future, we intend to establish the correlation between the amount of FMD vaccine antigen level and the results of VNT for each vaccine from different companies. For the VNT, we plan to conduct a field trial vaccination test in animals for vaccines from different companies. In this approach, we just require a mutual correlation value between antigen content measured by HPLC and antibody titer by VNT. That is, antigen values do not represent absolute 146S antigen content, rather relative measurements thereof produced by HPLC in the APQA lab. If the VNT can be replaced with antigen content determined by HPLC, it can efficiently reduce animal purchase expense, contribute to animal health and welfare, and reduce the assay time from 1 month to only 2 days. It can also accomplish standardization of quality assessment for all FMD vaccine products.

According to the World Organisation for Animal Health (OIE) terrestrial manual [14], FMD vaccines can be classified as either standard or higher potency vaccines. Standard potency vaccines are formulated to contain sufficient antigen and an appropriate adjuvant to ensure that they meet the minimum potency level required [recommended 3 PD_50_ (50% protective dose)] for the duration of the shelf life claimed by the company. This kind of vaccine is generally suitable for use in routine vaccination campaigns. For vaccination in naïve populations to control FMD outbreaks, higher potency vaccines (for instance >6 PD_50_ for the duration of the shelf life claimed by the company) are recommended for their wider spectrum of immunity and their rapid onset of protection. Thus, each vaccine company has their own correlation result between VNT value and PD_50_. However, most companies do not disclose the antigen content to the public. This antigen information is normally confidential. Therefore, we do not know the exact amount of each FMD vaccine. Here, with the exception of C1, the other companies did not provide the antigen content in the FMD vaccine. Furthermore, a high antigen content in a vaccine is not essential for potency. For instance, the formulations with a low antigen content with good adjuvant are sufficient to protect against FMD with 3 PD_50_. In Korea, vaccines from all three companies are officially approved by the Korean Government Authority, and the PD_50_ of these vaccines is more than 6, which is a requirement set by the Korean Government Authority. Although vaccines from the three different companies have different antigen content for each serotype, they exhibit satisfactory potency in animals.

The validation steps performed in the present study are necessary for the establishment of tests for national lot releases of the FMD vaccine. The effectiveness of the FMD analysis method was verified in terms of specificity, linearity, accuracy, repeatability, LOD, LOQ, and intermediate precision. Values for each parameter were in accordance with the World Health Organization (WHO) [17], OIE [18], and International Conference on Harmonisation (ICH) guidelines [19]. The present results validate the use of the HPLC technique for the measurement of FMD antigen content in FMD vaccines. Vaccines of the same batch from each company were also validated for analytical repeatability (three trials were performed for each same vaccine batch), similar to the standard material. These validation results also showed a good %CV value (<3.0%) for each vaccine batch.

In general, each manufacturer applied each method to measure antigen content for quality control. For instance, in M1, antigen separation was performed by chloroform followed by the digestion of aqueous phase samples with Benzonase. M2 used butanol for antigen separation followed by aqueous phase quantification by enzyme-linked immunosorbent assay. In M3, we used n-pentanol and trichloroethylene for separation followed by aqueous phase measurement by the sucrose density gradient analysis. Potency test methods differ among FMD vaccine companies; therefore, it is important to simplify the quality control procedure. Therefore, we evaluated three antigen separation methods for FMD vaccines from three manufacturers.

We evaluated the antigen content in the FMD vaccines from each manufacturer by HPLC. Each of the manufacturers recommends a different method, all of which required modifications for HPLC assays in this study. We applied a modified nuclease digestion protocol following each method using Benzonase endonuclease to remove DNA and RNA [20]. Owing to the similarity in the apparent hydrodynamic radius between the host cell DNA and the virus, an undigested sample would result in only partially resolved peaks. Benzonase cuts DNA into smaller molecules for subsequent separation by chromatography.

We determined the optimal separation method for assessing the antigen content in different FMD vaccines by HPLC. The quantitation of FMD virus antigens in vaccines by HPLC has been validated by Biogénesis Bagó S.A. [12]. We validated the HPLC method independently using a standard material to measure vaccines in Korea and confirmed that this method provides rapid, simple, precise, and accurate measurements. As several FMD vaccines are imported, a general testing method applicable to all vaccine types is urgently needed. The antigen measurement methods provided by the manufacturers were all time-consuming and required modifications. We established an optimized protocol for antigen measurement by HPLC; however, an appropriate separation method is necessary to yield accurate results.

Normally, vaccines from different batches have different antigen contents and potency values of VNT or PD_50_. However, all vaccines meet the potency cutoff of VNT or PD_50_. Therefore, it is necessary to understand the variation effect of different batch vaccines from each serotype and company. We found that the HPLC method was applicable to all batches of vaccine and we obtained the mean antigen content for all batches of vaccines. Our final goal is to compare between antigen content and VNT value of each serotype and company. Therefore, we require more information of different batches of vaccines, including antigen content and VNT value; the average results can be used to analyze the relationship between antigen content and VNT value. For vaccines produced by C1, C2, and C3, M3 was the most effective method, and there was little difference between M1 and M2. Overall, M3 yielded the highest antigen content for all vaccines, regardless of the manufacturer or serotype, even for those with low antigen content. M3 was also the most effective separation method for HPLC after treatment with Benzonase. 

In this study, we propose SE-HPLC as a viable measurement tool for various FMD vaccines, after a modified separation method. Of note, the values of each vaccine do not represent absolute 146S antigen content, but relative measurements obtained by HPLC in the APQA laboratory. The 146S quantitative sucrose density gradient analysis was developed in 1974 [21]. For more than four decades, this method has been used to measure virus concentrations. This general approach is considered the ‘gold standard’ for antigenic content determination in FMDV manufacturing and vaccine quality control, despite several limitations [12]. In a previous study, a validated HPLC method was reported as a suitable replacement for the sucrose density gradient analysis of 146S procedures for both in-process control of FMDV vaccine manufacturing and the final vaccine control by research institutions and regulatory agencies, with substantial advantages in terms of accuracy, precision, and automation capabilities [12]. Because C1 officially reported the antigen content in vaccines, we also tested three different vaccine batches to confirm the antigen content by sucrose density gradient analysis. The results were consistent and showed a good correlation coefficient value (0.9773) with the values obtained by HPLC for M3-separated vaccines, and thus, we confirmed that both sucrose density gradient and HPLC methods are effective for the analyses of antigen content in FMD vaccines, similar to the findings of a previous study [12]. Additionally, the detected recovery rate of C1 vaccine obtained by both HPLC and the sucrose density gradient was the highest for M3-separated vaccines. Although recovery rates obtained by HPLC for M3-separated vaccines were more than 100%, antigen values do not represent absolute 146S antigen content, rather relative measurements produced by HPLC in the APQA lab. The antigen contents and recovery rates obtained by HPLC were slightly (mean 20%) higher than those obtained by sucrose density gradient. In a previous study, a slight increase (~20%) in antigen contents was also observed for vaccine aqueous phases and was attributed to the spread of some viral contents along the sucrose density gradient, as detected by western blot of viral proteins in gradient fractions, whereas HPLC elutes displayed a western blot signal only in the virus peak [12].

Currently, in Korea, the potency of the FMD vaccine is determined by quantifying antibodies using the VNT after animal vaccination. Our goal was to evaluate the quality of various FMD vaccines by SE-HPLC, thereby eliminating the need for VNTs of animal serum. Based on our results, we recommend quantitative FMD 146S antigen detection by HPLC and comparisons against the VNT values from animal serum. If the values are correlated, HPLC alone would be sufficient for quality control analyses. The measurement of antigen content by HPLC may improve the quality control of FMD vaccines. This method also resolves animal welfare concerns by reducing animal experiments and saves money and time. 

## 4. Materials and Methods 

### 4.1. Standard

A high concentration standard was provided by Biogénesis Bagó S.A. The standard was a highly purified, inactivated FMD virus containing concentrated 146S antigen of the O1 Campos strain. 

### 4.2. Validation of Test Methods

As a part of the transfer process from Argentina to South Korea, and to assess suitability of the method for national lot-release testing, the specificity, linearity, repeatability, accuracy, LOD, and LOQ were evaluated per the WHO [17], OIE [18], and ICH guidelines [19].

### 4.3. Vaccines

Inactivated commercial FMD virus vaccines were imported from three companies, referred to as C1, C2, and C3. All vaccine antigens were derived from O or A serotype of the FMD virus. C1 produces a vaccine against serotype O+A+A, C2 produces vaccines against serotypes O, A, and O+A, and C3 produces a vaccine against serotype O+O+A. C1 used a W/O emulsion. C2 and C3 used a W/O/W emulsion.

### 4.4. Vaccine Pretreatment

Each manufacturer provided a separation method for antigen isolation from the aqueous phase of oil emulsion vaccines after reagent treatment. In M1, the vaccines were thoroughly mixed with the same volume of chloroform (Merck KGaA, Darmstadt, Germany) and centrifuged, followed by careful decantation; this process was then repeated to obtain pure antigen, and the final upper aqueous phase was used for quantification. In M2, 8% 1-butanol (Merck KGaA) was added to vaccine sample and mixed by vortexing (Scientific Industries, Inc., Bihemia, NY, USA), and then the sample was allowed to rest. This procedure was repeated three times, followed by centrifugation; the lower aqueous phase was then collected for antigen quantification. In M3, vaccine sample was mixed with chilled n-pentanol at 4 ℃ (Sigma-Aldrich, St. Louis, MO, USA) and maintained at 4 ℃ for 1 h. After layer separation, the lower aqueous phase was collected. Trichloroethylene (Merck KGaA) was added to the separated phase, then the sample was mixed vigorously and centrifuged. The upper aqueous phase containing the FMDV was used for quantification. For HPLC, the final samples obtained from the three methods were subjected to nuclease digestion.

### 4.5. Nuclease Digestion for Chromatography

Benzonase (Merck KGaA) was used for host cell DNA digestion after separation. A working solution of 1.25 units/µL concentration was prepared with 200-fold diluted enzyme in the Benzonase dilution buffer (50 mM Tris, 20 mM NaCl, and 2 mM MgCl_2_). For the three separation methods (M1–M3), 1 mL of sample was mixed with 20 μL of Benzonase working solution, incubated at 37 ℃, and mixed at 1400 rpm for 1 h in an Eppendorf Thermomixer (Eppendorf AG, Hamburg, Germany) for digestion. After centrifuging at 16 000 × *g* and 4 ℃ for 10 min, 900 μL of each treated sample was transferred to a vial for HPLC [11].

### 4.6. Size Exclusion HPLC

Size exclusion (SE)-HPLC was performed on the Agilent 1260 Infinity Binary LC (Agilent, Santa Clara, CA, USA) at the Veterinary Drugs and Biologics Division of the APQA. The LC was equipped with a G1311C quaternary pump with degasser and a G4212B variable wavelength detector with UV monitoring at 254 nm. The chromatograph was fitted with the TSKgel G4000 PWXL analysis column (7.8 mm inner diameter × 30 cm; Tosoh Corporation, Bioscience Division, Tokyo, Japan) and the TSKgel PWX guard column (6.0 mm inner diameter × 4.0 cm, Tosoh Corporation, Bioscience Division, Tokyo, Japan) to extend column life. OpenLab Chromatography data system (CDS) ChemStation Edition Rev. C01.02 was used for analysis. The mobile phase consisted of a mixture of 30 mM Tris-HCl and 400 mM NaCl adjusted to pH 8.0 with hydrochloric acid. Hundred microliters of each sample was injected and eluted via isocratic flow at 0.5 mL/min in the mobile phase. The operating pressure of the HPLC was approximately 40 bars at a column oven temperature of 20 ℃.

### 4.7. Calculations

The formula provided by Biogénesis Bagó FMD (Argentina) [10] was used to quantify antigen content by measuring the peak area as follows (Equation (1)):

(1) $$Virus\;concentration\;\left[\frac{\mu g}{mL}\right]=\frac{Area\;\left[mAU*s\right]\times 10\times 1.02}{120\times 72\times Injection\;volume\;\left[\mathrm{mL}\right]}$$

where Area is the peak area, obtained using OpenLab CDS ChemStation in mAU*s; Injection volume is the sample volume (in mL) injected into the column; 120 is the number of seconds required for 1 mL of mobile phase to flow through the detector cell; 72 is the published [21] specific absorptivity of the FMDV, as used in the 146S reference method, for a 1-cm optical path cell; 1.02 is a correction factor that accounts for 20 μL of Benzonase working solution added to 1000 μL of sample; and 10 is a unit conversion factor.

### 4.8. Sucrose Gradient Centrifugation 

Each treated vaccine sample from C1 was subjected to sucrose gradient ultracentrifugation as described previously [10,21] for analytical purpose.

### 4.9. Correlation Coefficient Analysis with Sucrose Gradient Centrifugation

Results obtained with the HPLC method were compared to those obtained by the standard 146S sucrose gradient ultracentrifugation method for FMDV antigen content determination. In C1 company, a total of three different batch vaccines obtained with M3 were analyzed both by HPLC and by the 146S method. We used the correlation coefficient method.

## Figures and Tables

**Table 1:** Repeatability and recovery rates for the FMD virus by the HPLC method.

Standard Sample (μg/mL)	Detected Amount (μg/mL)	Recovery Rate (%)	Coefficient of Variation (%)
73	66.6	91.2	0.28
	66.42	91.0	
	66.23	90.7	
58.4	52.84	90.5	0.62
	52.7	90.2	
	52.22	89.4	
43.8	39.74	90.7	0.57
	39.53	90.3	
	39.29	89.7	
29.2	26.25	89.9	0.89
	26.16	89.6	
	25.81	88.4	
14.6	12.88	88.2	1.11
	12.7	87.0	
	12.6	86.3	

**Table 2:** Precision of HPLC method for virus detection.

Standard Sample (μg/mL)	Intra-day Assay (n = 3)		Inter-day Assay (n = 3)	
	Average Amount (μg/mL)	Coefficient of Variation (%)	Average Amount (μg/mL)	Coefficient of Variation (%)
73	66.4 ± 0.2	0.28	65.2 ± 1.4	2.08
58.4	52.6 ± 0.3	0.62	51.3 ± 1.5	2.89
43.8	39.5 ± 0.2	0.57	38.3 ± 1.3	3.51
29.2	26.1 ± 0.2	0.89	25.2 ± 1.0	3.94
14.6	12.7 ± 0.1	1.11	12.3 ± 0.5	3.80

**Table 3:** Limit of detection (LOD) and limit of quantification (LOQ) of the HPLC method.

Calibration Curve Parameter	LOD (μg/mL)	LOQ (μg/mL)
*y* = 77.684*x* - 59.587 (*r^2^* = 0.9999)	0.5734	1.7377

**Table 4:** Summary of parameters of the HPLC analysis method for the FMD virus.

Analytical Conditions	Results
Column	TOSOH TSKgel G4000 PWXL Bioscience (7.8 mm inner diameter × 30.3 cm L)
Mobile phase	Tris-HCl:NaCl
Maximum pressure	40 bar
Run time	58 min
Flow rate	0.5 mL/min
Absorbance	254 nm

**Table 5:** Comparison of antigen content and recovery rate determined by HPLC and sucrose density gradient in vaccines (different batches), from C1 companies (serotype O+A+A), separated by three different methods.

Method	HPLC					Sucrose Density Gradient				
	Average Antigen Content (μg/mL)	CV (%)	Recovery Rate (%)	Average Amount (μg/mL)	SD	Average Antigen Content (μg/mL)	CV (%)	Recovery Rate (%)	Average Amount (μg/mL)	SD
M1	24.8	2.0	49.4	30.3	4.2	26.4	2.1	52.7	34.8	6.9
	33.2	2.1	59.9			42.1	1.6	76.0		
	32.9	1.2	56.7			35.9	1.3	61.8		
M2	22.8	2.4	45.4	22.7	4.5	26.5	1.7	53.0	25.2	4.3
	27.9	2.2	50.4			29.4	2.1	53.0		
	17.5	2.3	30.2			19.8	2.5	34.1		
M3	51.2	1.2	102.2	57.9	5.3	39.8	1.0	79.4	46.8	5.3
	59.3	1.0	107.0			49.7	0.9	89.7		
	63.1	0.7	108.9			51.0	0.6	88.0		

CV, Coefficient of variation; SD, Standard deviation; M1, chloroform; M2, butanol; M3, n-pentanol and trichloroethylene.

**Table 6:** Comparison of antigen content determined by HPLC in vaccines (different batches) from two companies separated by three different methods.

Company	Serotype	Method	Antigen Content (μg/mL)	CV (%)	Average Amount (μg/mL)	SD
C2	O	M1	2.2	2.3	3.2	1.2
			4.3	2.3		
		M2	1.9	2.8	3.3	1.6
			4.8	2.1		
		M3	5.1	1.1	6.7	1.7
			8.2	1.2		
	A	M1	12.2	2.1	12.4	0.3
			12.5	2.1		
		M2	12.0	2.1	12.1	0.2
			12.1	2.2		
		M3	13.8	1.9	14.0	0.3
			14.1	1.8		
	O+A	M1	19.6	1.8	20.5	2.7
			20.6	2.4		
			17.5	2.2		
			24.4	2.1		
		M2	18.2	2.3	18.2	1.5
			17.1	2.4		
			17.0	2.1		
			20.5	2.2		
		M3	23.1	1.1	25.8	3.0
			23.0	1.3		
			29.5	1.4		
			27.7	1.3		
C3	O+O+A	M1	2.4	1.3	2.7	0.3
			2.6	1.7		
			3.0	2.3		
			2.2	2.6		
			2.9	2.0		
			3.0	2.4		
			2.4	2.5		
		M2	1.7	2.7	2.2	0.5
			1.7	2.7		
			2.0	2.8		
			3.2	2.8		
			2.2	2.7		
			2.5	2.7		
			1.6	2.8		
		M3	5.3	1.6	7.1	1.3
			6.6	1.3		
			8.6	1.1		
			6.5	1.3		
			8.3	1.0		
			8.3	1.0		
			5.8	1.2		

SD, Standard deviation; M1, chloroform; M2, butanol; M3, n-pentanol and trichloroethylene.
